# Preliminary research indicates that mechanical force through Pioze1 enhances local immunity during NPWT treatment for spinal infections

**DOI:** 10.3389/fimmu.2025.1600194

**Published:** 2025-06-03

**Authors:** Hao Xing, Junlin Pan, Huan Liu, Yanan Wang, Zhengqi Chang

**Affiliations:** ^1^ Department of Orthopaedics, The 960th Hospital of People’s Liberation Army of China (PLA), Jinan, China; ^2^ Department of Reproductive Medicine, The 960th Hospital of People's Liberation Army of China (PLA), Jinan, China

**Keywords:** spinal infection, negative pressure wound therapy, Piezo1, mechanical force, immune response

## Abstract

**Objective:**

This study aims to investigate the mechanism by which Negative Pressure Wound Therapy (NPWT) regulates local immune responses in spinal infection through Piezo1-mediated mechanical stress, and elucidate its potential role in the treatment of spinal infections.

**Methods:**

From July 2021 to April 2022, a total of 7 patients with spinal infection treated with NPWT at our department were included in the study. The study analyzed clinical outcomes of spinal infection surgeries, including operative duration, intraoperative blood loss, postoperative drainage, improvements in pain levels as measured by the Visual Analogue Scale (VAS), and inflammatory markers such as C-reactive protein (CRP) and Erythrocyte Sedimentation Rate (ESR) measured one week before and after the procedure. Additionally, healing times and recurrence rates within two years post-surgery were assessed. In addition, lesion specimens were retained during surgery and changes in Piezo1, Interleukin-1β (IL-1β), IL-6, IL-8, and Tumor Necrosis Factor-α (TNF-α) in lesion tissues were observed before and after immunohistochemical analysis.

**Results:**

All 7 patients with spinal infections successfully underwent NPWT treatment and were ultimately cured. The average healing time was 45.71 ± 9.49 days, and there were no cases of recurrence or death during the two-year follow-up period. Surgical data showed a surgery duration of 96.57 ± 13.31 minutes, intraoperative blood loss of 65.71 ± 29.36 milliliters, and postoperative drainage of 163.57 ± 11.07 milliliters. Postoperatively, CRP, ESR, and VAS all significantly improved compared to preoperative levels (all p<0.05), which was superior to traditional treatment methods. Following NPWT intervention, the expression of Piezo1 protein at the lesion site significantly increased (0.03 ± 0.11 vs. 0.27 ± 0.22; p<0.05), while the expression levels of IL-1β, IL-6, IL-8, and TNF-α in the local immune microenvironment of the infected lesion significantly decreased (0.26 ± 0.11 vs. 0.16 ± 0.09, 0.27 ± 0.12 vs. 0.15 ± 0.67, 0.26 ± 0.18 vs. 0.10 ± 0.12, 0.35 ± 0.21 vs. 0.15 ± 0.11; p<0.05).

**Conclusion:**

Clinical results demonstrate that NPWT treatment for spinal infections exhibits remarkable efficacy, accompanied by a notable augmentation in local Piezo1 protein consistency. It is hypothesized that the mechanical force employed in NPWT treatment stimulates the Piezo1 protein, thereby modulating local immune cells and factors, ultimately bolstering local immunity. This study not only provides a molecular biology basis for a deeper understanding of the therapeutic effects of NPWT, but also offers new insights for optimizing treatment strategies for spinal infections.

## 1 Introduction

With the arrival of an aging population, the incidence of bone and soft tissue infections is increasing, with spinal infections being common (1–3). Kehrer found that the incidence of spinal infections has increased dramatically to 2.2-5.8/100000 since 2000 (4). If spinal infections are not taken seriously or if treatment is ineffective, it can easily lead to the spread of infection and the formation of abscesses in the spine, causing serious complications such as nerve root or spinal cord compression and neurological dysfunction. Current surgical approaches often advocate for one-stage posterior lesion clearance combined with bone graft fusion and internal fixation, but they have drawbacks such as high trauma, low cure rates, and high mortality rates (5). Studies have shown that if the infection is not completely cleared from the body, it can lead to the continuous production of pro-inflammatory cytokines such as IL-1β, IL-6, IL-8, and TNF-α, which can limit the formation of granulation tissue and microvessels (6).

In recent years, multiple randomized controlled trials have highlighted the significant advantages of NPWT in treating infected wounds. NPWT is a sealed dressing system that applies mechanical stress to the wound, promoting healing through various biological mechanisms (7, 8). Piezo1, a mechanosensitive, non-selective cation channel, plays a pivotal role in transmitting mechanical-chemical signals that regulate cellular responses to external mechanical stress (9). Previous clinical evidence has demonstrated the benefits of NPWT in treating spinal infections, including promoting neutrophil chemotaxis to control infection spread and improving systemic inflammatory markers (10, 11). The primary mechanisms by which NPWT aids in treating infected wounds involve enhancing microvascular formation, stimulating granulation tissue generation, reducing bacterial load, and alleviating inflammation and edema (12–14). However, research on the impact of NPWT on the local immune microenvironment in spinal infections remains limited. Therefore, this study aims to further investigate the mechanisms underlying NPWT’s regulation of the local immune response in spinal infections, focusing on Piezo1-mediated mechanical stress and its effects on local inflammatory factors, with the goal of providing a more comprehensive theoretical foundation for the clinical application of this therapy.

## 2 Materials and methods

### 2.1 Standards for inclusion and exclusion, follow-up time and follow-up loss rate

This study was conducted from July 2021 to April 2022, with a total of 7 patients included. Inclusion criteria were: (1) Patients aged ≥18 years diagnosed with spinal infection through medical history, imaging, and pathology; (2) Patients receiving NPWT during the study period and able to provide sufficient and complete pathological specimens before and after treatment. Exclusion criteria were: (1) Patients with postoperative wound infections due to spinal surgery; (2) Patients receiving immunosuppressive therapy (such as biologics, immunosuppressive drugs, etc.), corticosteroids, or chemotherapy; (3) Patients with severe heart, kidney, liver, or other organ dysfunction, or other acute critical illnesses; (4) Patients refusing NPWT treatment for personal reasons or other factors. All patients will be followed up for at least 24 months after treatment. Patients who fail to attend follow-up appointments as scheduled and cannot be contacted by phone or other means will be considered lost to follow-up. From July 2021 to April 2022, our team treated a total of 21 patients with spinal infections, with 9 cases not receiving NPWT treatment and 12 cases receiving NPWT treatment. Out of these, 7 cases with pre- and post-operative pathological specimens were included in the study.

### 2.2 Data collection

Collect information on patient gender, age, diagnosis, pathogenic microorganisms, as well as surgical time, intraoperative blood loss, postoperative drainage volume, pre- and post-operative improvement in VAS, CRP, ESR, healing time, and 2-year recurrence rate. Additionally, obtain tissue samples from the infected lesion before and after NPWT treatment for spinal infection, and analyze the expression changes of Piezo1, IL-1β, IL-6, IL-8, and TNF-α in the lesion tissue using hematoxylin-eosin staining and immunohistochemical analysis.

### 2.3 Surgical procedure

**Figure 1:**
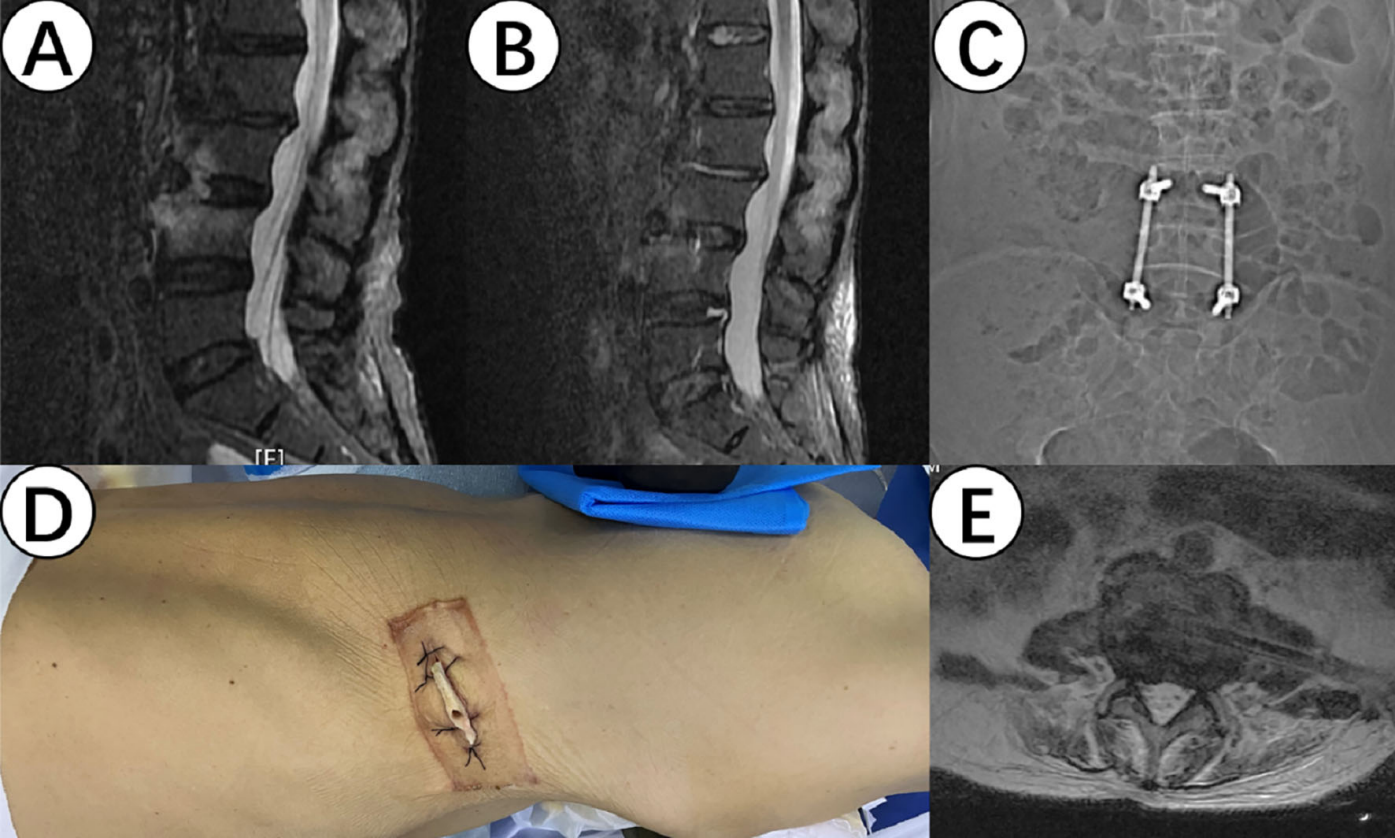
**(A, B)** show the magnetic resonance imaging (MRI) results of the lesion site before and after surgery, demonstrating a significant improvement in inflammatory signals. **(C)** shows internal fixation for intersegmental fixation to prevent lumbar instability and collapse. **(D)** shows the implantation of a drainage sponge through an extremely lateral approach. **(E)** shows the position of the drainage sponge in the intervertebral gap on the MRI.

All 7 patients with spinal infections underwent NPWT treatment during surgery. Sterile polyvinyl alcohol foam dressings were used intraoperatively, tailored to fit the size of the intervertebral infection wound. The dressings extended to the skin surface while another dressing covered the surgical incision on the skin surface, followed by a polyurethane medical dressing to create a sealed microenvironment. The foam dressings included a suction tube extending to the outside of the body, connected to an extension tube leading to a negative pressure collection bottle, which was connected to an adjustable negative pressure source (-125mmHg) (15) (as shown in **Figure 1**). Patients underwent adequate pathological tissue sampling from the infection site intraoperatively and 7 days postoperatively.

### 2.4 Immunohistochemical analysis

**Figure 2:**
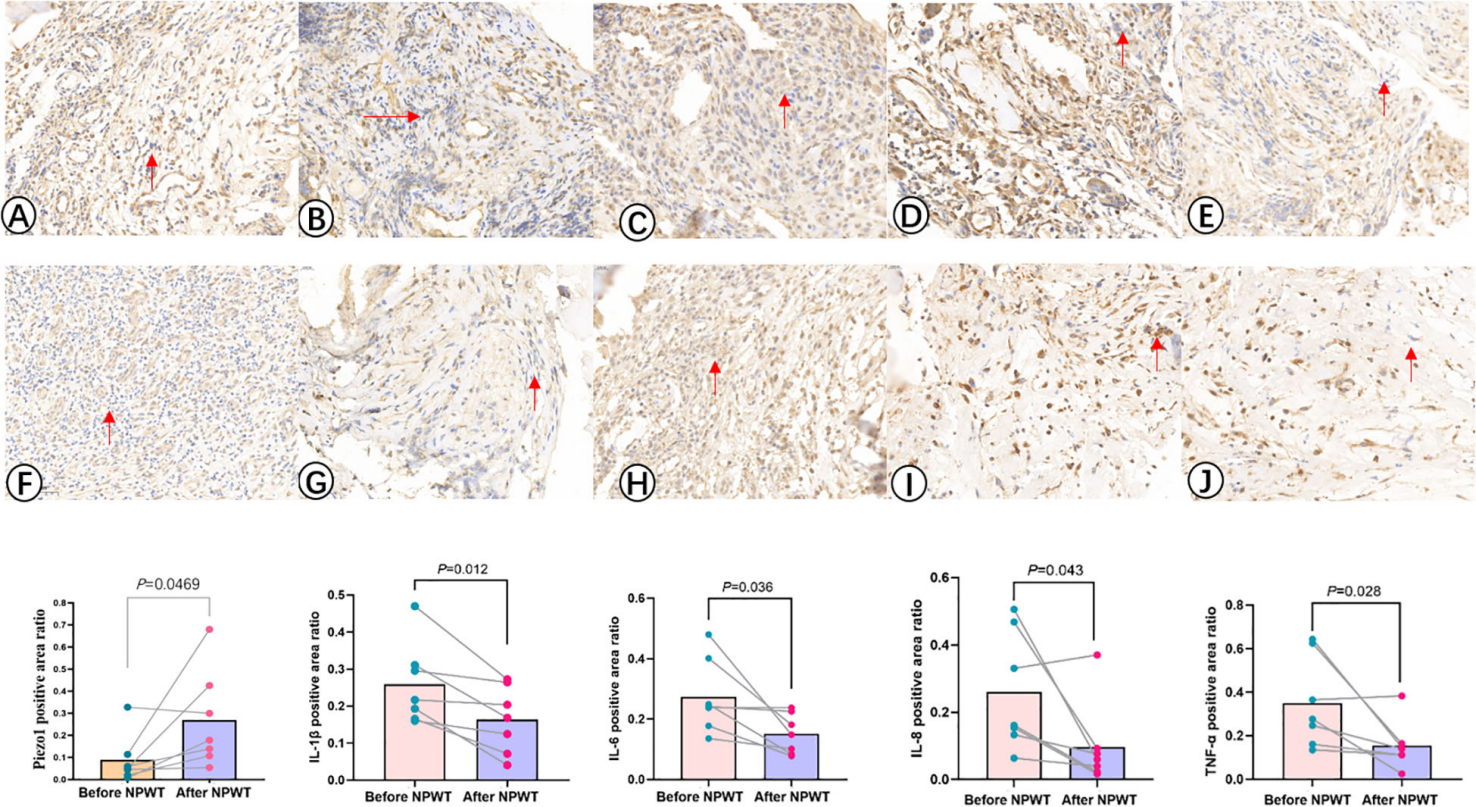
Immunohistochemistry images illustrate the expression levels of Piezo1, IL-1β, IL-6, IL-8, and TNF -α before **(A–E)** and after **(F–J)** NPWT treatment. The statistical result reveals a notable increase in the expression of Piezo1 after the treatment, along with a significant decrease in the expression of IL-1β, IL-6, IL-8, and TNF-α following NPWT intervention.

Immunohistochemical analysis method was used to detect the expression of Piezo1, IL-1β, IL-6, IL-8, and TNF-α. Tissue samples were fixed in 10% buffered formalin, embedded in paraffin, and cut into standard 3μm sections using a pathological slicer (Shanghai Leica Instrument Co., RM2016). The sections were deparaffinized, underwent antigen retrieval, blocked endogenous peroxidase, and then blocked with Bovine Serum Albumin (BSA). Primary antibodies were incubated overnight at 4°C, followed by secondary antibodies incubated at 37°C for 50 minutes. Finally, the sections were counterstained with hematoxylin to stain the cell nuclei, dehydrated, and coverslipped (**Figure 2**). The immunohistochemistry slides were analyzed using Aipathwell digital pathology image analysis software (Servicebio^®^), and the positive staining areas were recorded.

### 2.5 Statistical analysis

Statistical analysis was conducted using SPSS 21.0 software. Descriptive statistics for continuous data were presented as mean ± standard deviation, and normality and homogeneity of variance tests were performed. For normally distributed data, paired sample t-tests were used for comparison between the two groups; for non-normally distributed data, the Wilcoxon signed-rank test was used. A significance level of P < 0.05 was considered statistically significant.

## 3 Results

Among 7 patients diagnosed with spinal infections, 5 were male and 2 were female, with an average age of 52.7 ± 13.0 years (ranging from 38 to 72 years). The affected spinal levels were L2/3 in 1 case, L3/4 in 3 cases, and L4/5 in 3 cases. Following NPWT treatment, there was an improvement in systemic inflammatory markers such as CRP and ESR. Moreover, there was a significant short-term improvement in VAS (7.00 ± 0.82 Vs. 2.29 ± 0.76, P<0.05). The average time for complete healing was 45.71 ± 9.49 days, and there were no instances of recurrence or mortality during the 2-year postoperative follow-up. Surgical data revealed an average operation duration of 96.57 ± 13.31 mins, intraoperative blood loss of 65.71 ± 29.36ml, and postoperative drainage of 163.57 ± 11.07ml (**Table 1**).

**Table 1:** Baseline data of 7 patients.

Number	Sex	Age (years)	Lesion location	Bacteria	Time of operation(mins)	Intraoperative blood loss(ml)	Postoperative drainage 5 days(ml)	Healing time (days)	Recurrence within 2 years
1	male	41	L4/5	Brucella	86	100	165	43	No
2	female	64	L4/5	Brucella	110	20	155	46	No
3	male	42	L4/5	Staphylococcus aureus	105	50	160	52	No
4	male	72	L3/4	Viridans Streptococci	110	50	155	52	No
5	male	59	L3/4	Enterobacter cloacae	100	60	155	59	No
6	male	53	L3/4	Brucella	75	100	185	33	No
7	female	38	L2/3	Mycobacterium Tuberculosis	90	80	170	35	No

L, lumbar.

The immunohistochemical analysis of Piezo1 and inflammatory factors in the infection lesions before and after treatment is shown in **Table 2**, **Figure 2**. The results of immunohistochemical examination suggest a significant decrease in inflammatory factors in the infected tissues after NPWT treatment, with a statistically significant difference.

**Table 2:** Comparison of related detection and treatment before and after NPWT for spinal intervertebral space infection (7 cases).

Group	CRP(mg/L)	ESR(mm/h)	Piezo1	IL-1β	IL-6	IL-8	TNF-α
Before NPWT	71.51±51.39	62.43±27.46	0.03±0.11	0.25±0.11	0.27±0.12	0.26±0.18	0.35±0.21
After NPWT	11.88±7.72	31.14±23.01	0.27±0.22	0.16±0.09	0.15±0.07	0.10±0.12	0.15±0.11
Statistical value	-2.366^b^	2.926^a^	-2.108^b^	3.544^a^	2.683^a^	-2.082^b^	-2.197^b^
P	0.018	0.026	0.047	0.012	0.036	0.043	0.028

a-T test, b- Non-parametric rank sum test; The values of Piezo1, IL-1β, IL-6, IL-8 and TNF-α represent the proportion of positive regions in the staining results.

## 4 Discussion

Spinal infection, as a special type of bone infection, has a higher recurrence rate and mortality rate compared to other bone infections, with reported recurrence rates of 10% and mortality rates reaching 20% (5). The main drawback of traditional treatment approaches is the inadequate blood supply to tissues, which significantly reduces the effectiveness of antibiotics. Additionally, the specific location of the wound also makes complete debridement challenging. NPWT has proven effective in promoting the healing of various complex wounds, such as extensive burns, postoperative incision infections, necrotizing fasciitis, and skin graft sites (16–19). Several randomized controlled trials have demonstrated that NPWT offers significant benefits in treating infected wounds by reducing local inflammation and edema, stimulating granulation tissue and microvascular formation, and lowering bacterial load (7, 8, 12–14). Our team has achieved favorable clinical outcomes using NPWT to treat spinal infections (10, 11). Pappalardo et al. (20) clearly highlighted the safety and effectiveness of NPWT in managing post-spinal surgery infections through a systematic review. In this study, all seven patients who received NPWT treatment experienced positive clinical outcomes, with an average healing time of 45.71 ± 9.49 days and no recurrence or mortality during the two-year follow-up period. Furthermore, all patients showed significant improvements in postoperative VAS scores, CRP, and ESR within a short period. Our clinical findings indicate that NPWT treatment is significantly more effective than traditional methods, consistent with reports in the literature. This suggests that NPWT not only facilitates drainage but also offers therapeutic benefits.

In addition to draining, NPWT also exerts mechanical force through negative pressure suction. Nonetheless, mechanical physical signals, in contrast to soluble chemical signals like cytokines, are less investigated as regulators of immune cell function. Atcha et al. (21) found that physical mechanical stress and soluble signals synergistically regulate macrophage morphology and function, proposing that the crosstalk between CD11b and Piezo1 plays a crucial role in mechanical transduction in macrophages. Orsini et al. (22) discussed the role of mechanical sensitive channels such as Piezo1 in immune cells under various physiological and pathological conditions. They believe that mechanical signals can activate various signaling pathways within cells through the activation of mechanosensitive ion channels and receptors, including MAPKs, YAP/TAZ, EDN1, NF-kB, and HIF-1a, thereby altering the physiological state of the cell. Mulhall et al. (23) used nanoscale fluorescence imaging technology to directly observe the conformational changes of Piezo1 within the cell membrane, demonstrating its direct response to mechanical stress. Following mechanical stimulation, Piezo1 enhances integrin β1 (ITGB1) activity, forming the Piezo1/ITGB1 signaling axis, which triggers cell cytoskeletal reorganization through the RhoA/ROCK pathway, thereby enhancing Piezo1’s mechanosensitivity and establishing a positive feedback loop (24, 25). Chen et al. (9) found that high blood flow shear stress activates Piezo1, leading to the activation of various inflammatory signaling pathways such as NF-κB in a rat model of pulmonary arterial hypertension induced by left pulmonary artery ligation. This results in upregulation of Piezo1 expression in pulmonary vascular endothelial cells. Similarly, by comparing infected tissue samples before and after NPWT treatment, we found a significant increase in Piezo1 expression levels after NPWT treatment, suggesting that the mechanical stress provided by NPWT and high blood flow shear stress may both upregulate Piezo1 expression in cells.

IL-1β, IL-6, IL-8, and TNF-α are inflammatory factors that play important roles in the immune microenvironment during the entire immune response process. IL-1β is an inductive cytokine mainly produced by activating M1 macrophages (26). Tao et al. (27) found that NPWT treatment for diabetic foot infection significantly reduced IL-1β expression after 7 days of use. IL-6 has a wide range of effects in immune regulation, acute phase response, regeneration, and bone homeostasis (28). Wang et al. (29) found that NPWT can significantly reduce the expression levels of IL-6 in infected wounds of diabetic foot patients, inhibit chronic inflammation, and promote wound healing. IL-8 is a leukocyte-specific chemotactic cytokine that plays an important role in recruiting polymorphonuclear neutrophils in the early inflammatory stage of wound healing (30). TNF-α is a classical pro-inflammatory cytokine mainly produced by activated macrophages, playing a crucial role in immune responses in the immune microenvironment, including regulating inflammation, cell apoptosis, and immune cell proliferation. Norbury et al. found that the levels of TNF-α in wound drainage fluid decreased after NPWT treatment in experimental animals (31). This study focuses on the changes in the expression levels of inflammatory factors IL-1β, IL-6, IL-8, and TNF-α in infected local tissues. Continuous application of NPWT mechanical stress was found to decrease the expression of IL-1β, IL-6, IL-8, and TNF-α.

Research has shown a close association between Piezo1 and most inflammatory factors (32, 33). Piezo1, as a mechanosensitive ion channel protein, mediates calcium influx into immune cells such as macrophages, dendritic cells, and neutrophils under NPWT mechanical stress intervention, activating various downstream signaling pathways to elicit a series of immune response reactions. For example, Yang et al. found that Piezo1 activation enhances NF-κB pathway activity, promoting the production of inflammatory factors such as IL-1β, IL-6, IL-8, and TNF-α (34). However, in our clinical practice, we found that the expression levels of inflammatory factors in local tissues decreased after 7 days of NPWT mechanical stress intervention. This may be due to two reasons: (1) Piezo1 promotes wound healing by regulating the proliferation, migration, and collagen synthesis of fibroblasts, reducing continuous tissue damage and indirectly slowing down the duration and severity of the inflammatory response (35); (2) Piezo1 has a certain pro-angiogenic effect, which helps improve local blood circulation, reduce wound edema, and promote early wound healing (36).

During the early stages of normal healing, a temporary increase in inflammatory factors helps promote immune cells (such as neutrophils, macrophages, etc.) to accelerate the clearance of bacteria and tissue necrosis. Activation of Piezo1 can enhance local immune responses. However, in the late stages of healing, prolonged excessive presence of inflammatory cells and factors can lead to continued inflammation, hindering tissue repair and healing. Therefore, it is necessary to regulate inflammation appropriately to avoid excessive inflammation affecting the healing process. This study found that after 7 days of NPWT on spinal infection lesions, the expression of inflammatory factors such as IL-1β, IL-6, IL-8, and TNF-α in the local immune microenvironment significantly decreased, indicating that the spinal infection had gradually been controlled and entered the tissue repair stage. Therefore, it is speculated that NPWT can regulate local tissue immune changes, allowing various inflammatory factors to be produced, elevated, and decreased in an orderly manner, thereby promoting the clearance of local bacteria and tissue repair.

Although there is limited research on the mechanism of NPWT in treating spinal infections, we speculate that NPWT promotes healing by regulating the local immune microenvironment based on clinical and immunohistochemical analysis. Research has shown that inflammatory factors such as IL-1β, IL-6, IL-8, and TNF-α can induce and recruit various types of white blood cells to participate in immune responses, which may lead to tissue damage and affect the healing of infections if present long-term (26, 37, 38). Therefore, changes in the expression of inflammatory factors in the local immune microenvironment are closely related to the progression of infections. Piezo1, as a key protein that allows immune cells to receive mechanical signals and convert them into chemical signals, can sense the mechanical stress of NPWT and regulate immune response reactions in the local immune microenvironment of infections.

NPWT has the potential to enhance the local immune response in spinal infections through Piezo1 mediation. Clinical studies have shown that NPWT can improve outcomes, reduce healing times, and manage infection rates in patients with spinal infections. Immunohistochemical analysis has demonstrated an increase in Piezo1 protein expression levels and a significant decrease in inflammatory factors (IL-1β, IL-6, IL-8, TNF-α) in the immune microenvironment following NPWT treatment. This indicates that NPWT activates Piezo1 protein to regulate the immune response at the site of spinal infection, leading to inflammation control and tissue repair. However, further research involving cellular and animal experiments is necessary to fully understand the physiological functions of the Piezo1 signaling pathway. It is important to consider individual differences among patients that may impact treatment outcomes, and future studies should aim to include a larger sample size for more reliable conclusions.

## Data Availability

The original contributions presented in the study are included in the article/supplementary material. Further inquiries can be directed to the corresponding author.
